# Association between tamoxifen and incidence of osteoporosis in Korean patients with ductal carcinoma in situ

**DOI:** 10.3389/fonc.2023.1236188

**Published:** 2024-01-08

**Authors:** Dooreh Kim, Jooyoung Oh, Hye Sun Lee, Soyoung Jeon, Woo-Chan Park, Chang Ik Yoon

**Affiliations:** ^1^ Division of Breast Surgery, Department of Surgery, Seoul St Mary’s Hospital, College of Medicine, The Catholic University of Korea, Seoul, Republic of Korea; ^2^ Department of Psychiatry, Gangnam Severance Hospital, Yonsei University College of Medicine, Seoul, Republic of Korea; ^3^ Biostatistics Collaboration Unit, Yonsei University College of Medicine, Seoul, Republic of Korea

**Keywords:** DCIS, endocrine treatment, tamoxifen, osteoporosis, breast cancer

## Abstract

**Introduction:**

The partial estrogen-agonist action of tamoxifen on bone receptors has beneficial effects on bone mineral density. However, in premenopausal women, the use of tamoxifen causes systemic estrogen depletion, which has detrimental effects on bone health. We aim to investigate the association between tamoxifen and osteoporosis in the real world using data from a longitudinal nationwide cohort of Korean patients.

**Methods:**

Data were collected from the National Health Insurance claims database in South Korea. Osteoporosis was defined by diagnostic codes accompanying prescription data for osteoporosis. The cumulative incidence was analyzed by Kaplan–Meier survival curves and the risk factors were analyzed using a multivariable Cox proportional hazard regression model.

**Results:**

Between 2009 and 2015, of the 4,654 women with ductal carcinoma *in situ* (DCIS) without prior osteoporosis, 2,970 were prescribed tamoxifen and 1,684 were not. A total of 356 DCIS survivors were later diagnosed with osteoporosis during a median follow-up period of 84 months. In the overall population, tamoxifen was associated with a low risk of osteoporosis, before and after propensity matching adjusted for age, operation type, and comorbidities (before matching, hazard ratio [HR]=0.69, 95% confidence interval [CI]=0.559–0.851, p<0.001; after matching, HR=0.664, 95% CI=0.513–0.858, p=0.002). In the subgroup analysis, findings were consistent in postmenopausal women but were not evident in the younger age group.

**Conclusion:**

In a nationwide cohort study, a low risk of osteoporosis was associated with the use of tamoxifen. The protective effect of tamoxifen was more profound in older women and was not related to the incidence of osteoporosis in younger women.

## 1 Introduction

Bone health in breast cancer survivors is an important issue regarding quality of life (1). In breast cancer patients, anti-tumor therapies, especially aromatase inhibitor, are all directed to suppress the estrogen level and it rapidly leads to reduced bone density compared to in their age-matched peers (2). At least 5 years of endocrine therapy has a substantial impact on bone mineral density.

In one report, aromatase inhibitors (AIs) replaced tamoxifen in postmenopausal women with breast cancer and showed better efficacy in terms of recurrence-free survival (3). AIs suppress the physiologic level of estrogen by inhibiting peripheral aromatization into estrogen, thereby leading to accelerated bone loss (4–6). Many studies have reported the risk of bone loss and increased fracture risk with AIs, but few studies have focused on the effect of tamoxifen (7).

Tamoxifen, widely used in cases of hormone receptor-positive breast cancer, ductal carcinoma *in situ* (DCIS), and those at high risk of breast cancer (8, 9), is known to have a protective effect on bone health in postmenopausal women. In younger, premenopausal women, tamoxifen has the opposite effects, resulting in an elevated risk of pathologic fractures, as reported in a population-based cohort study of breast cancer patients (10). Very few epidemiologic studies have compared bone density between breast cancer survivors and cancer-free women (11, 12). Breast cancer survivors have significantly low bone mineral density and increased risk of osteopenia and osteoporosis. However, most studies have primarily focused on older, postmenopausal patients, and the cancer treatments have varied among the studies. Nonetheless, patients with *in situ* disease receive tamoxifen regardless of their age and menopausal status.

This study aims to investigate the incidence of osteoporosis in breast cancer patients according to the use of tamoxifen using national insurance claims data from a longitudinal observational nationwide population-based cohort.

## 2 Methods

The study was approved by the Korean National Health Insurance Service (NHIS) and the Health Insurance Review and Assessment Service (HIRA) as well as by the Catholic University of Korea Institutional Review Board (IRB) (local IRB number: KC22ZISI0340). Written consent was not needed as the study was retrospective and the processed data were anonymous.

### 2.1 Data source and study design

This nationwide population-based cohort included women with DCIS as the primary diagnosis (International Classification of Disease, 10^th^ revision [ICD-10]: D05). The enrollment period was between January 2007 and December 2021, and a 2-year wash-out period was used for any previous malignant disease. The HIRA collects data about general healthcare services, such as diagnoses, medical treatment, and medication prescriptions. Patients who redeemed the cost of at least two prescriptions after breast cancer diagnosis were defined as tamoxifen users (prescription code: 234501ATB and 234502ATB). Osteoporosis diagnosis was based on ICD-10 codes (M80, 81, and 82) and concomitant prescription data of osteoporosis that included risedronate, ibandronate, etidronate, pamidronate, alendronate, zoledronic acid, and denosumab (**Supplementary Table 1**).

In the screening phase, we extracted the data of women aged ≥20 years in the NHIS database. The screening procedure was identical to that used in a previous study (13). We included patients diagnosed with DCIS between January 2009 and December 2015 who had not visited a physician for any other malignancies (ICD-10 code: any C code) during the 2-year washout period between 2007 and 2008. To minimize misclassification errors, patients with DCIS were defined as patients with a surgery code within 1 year of receiving their DCIS diagnosis. The following surgery codes were considered: N7133, wide excision; N7134, wide excision of the axillary breast; N7136, wide excision with axillary surgery; N7137, wide excision without axillary surgery; N7138, total mastectomy with axillary surgery; and N7139, total mastectomy without axillary surgery. The date of enrollment was defined as the day of surgery for DCIS. The follow-up period was based on the date of enrollment. Enrolled patients were monitored for cataracts until 2021. We evaluated the risk of osteoporosis in patients with DCIS who received tamoxifen. The risk of developing osteoporosis was analyzed on the basis of only diagnosis or diagnosis and treatment. Women who had osteoporosis prior to DCIS and those who had been diagnosed within a year of DCIS diagnosis were excluded.

### 2.2 Outcomes and confounding variable definition

The primary endpoint of this study was the cumulative incidence of osteoporosis diagnosis and treatment after adjuvant tamoxifen administration in patients with DCIS. Secondary analysis included subgroup analyses according to age: <45, 45–55, and >55 years, representing pre-, peri-, and postmenopausal status.

The diagnostic data on the following comorbidities were also collected to determine the confounding variables: diabetes (ICD-10 codes: E10, E11, E12, E13, and E14), hypertension (I10), hyperlipidemia (E78), chronic obstructive pulmonary disease (COPD; J44), chronic kidney disease (CKD; N18), liver cirrhosis (K74 and K703), and heart failure (I50). Comorbidities were defined using these codes in the 2 years prior to the enrollment date.

### 2.3 Statistical analysis

The baseline demographic and clinical characteristics of the two groups were compared using a t-test and chi-square test. The cumulative cataract incidence rates in both groups were displayed using Kaplan–Meier curves and compared using the log-rank test. Cox proportional hazard models were used to determine the hazard ratios (HRs) and 95% confidence intervals (Cis) and ascertain the occurrence of cataracts after adjusting for confounding variables. We applied the enter method. Statistical significance was set at a two-sided p-value of less than 0.05. Randomization was performed using an algorithm in the SAS software program (version 9.4, SAS Institute, Cary, NC, USA). To minimize bias, an estimated propensity score was used to match patients with DCIS who received tamoxifen with those who did not. Each patient was assigned a propensity score reflecting the probability of receiving tamoxifen. This was calculated for each patient using logistic regression analysis with variables such as age, breast surgery, and comorbidities. A nearest-neighbor greedy algorithm was used to match patients using propensity scores without replacement. The matching algorithm first selected a patient who received tamoxifen and then selected a patient who did not receive tamoxifen with the closest propensity score to that of the first selected patient. Patients were matched in a 1:1 ratio within 0.1 caliper width. In addition, 1:1 propensity score matching was used to maximize the number of patients with breast cancer (14).

## 3 Results

### 3.1 Patient cohort

**Figure 1:**
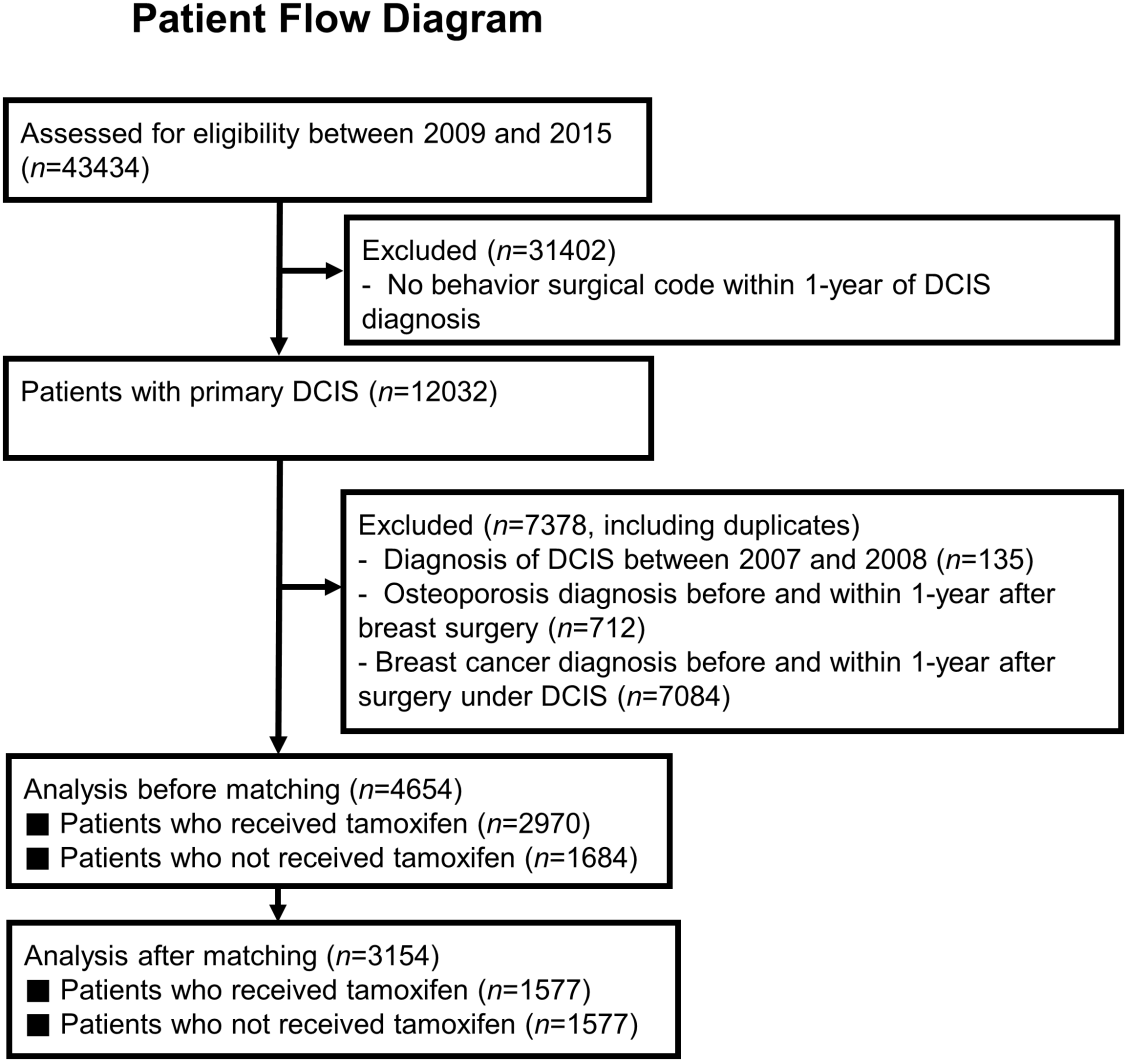
Patient flow diagram of study design.

Patients with diagnostic codes for DCIS between 2009 and 2015 were first sorted from the HIRA database. Out of a total of 43,434 patients with DCIS, 12,032 who had undergone curative breast surgery within 1 year after the first diagnosis were included. Patients who had not undergone curative breast surgery within 1 year were considered previously diagnosed and counted as duplicates (**Figure 1**). Patients who had co-existent diagnostic codes for invasive breast carcinoma or other types of malignancy were excluded. Further, 712 patients who were diagnosed with osteoporosis and prescribed treatment for osteoporosis before DCIS diagnosis were excluded from the main analysis. Among the remaining 4,654 patients, 2,970 were prescribed tamoxifen, and 1,684 were not prescribed any endocrine treatment. Propensity score matching was applied for age at diagnosis, chronic disorders, and type of breast surgery, as a surrogate for radiation therapy.

### 3.2 Demographics and incidence of osteoporosis

Before matching, the tamoxifen-treated and control groups consisted of 2,970 and 1,684 patients, respectively (**Table 1**). Among them, 356 (7.6%) patients were diagnosed with osteoporosis and were taking osteoporosis medication. After matching, the tamoxifen-treated and control groups consisted of 1,577 patients each (**Table 1**). Of the 3154 patients in the matched group, osteoporosis occurred in 250 (7.9%). **Table 1** shows the characteristics of the two groups in terms of chronic disorders, breast surgery type, and age. Before matching, the median age of the patients was 49 years. Patients who received tamoxifen were less likely to have osteoporosis than the control group (6.4% [191/2,970] vs. 9.8% [165/1,684], p<0.001). The tamoxifen group was slightly older and more likely to receive breast-conserving surgeries. In addition, diabetes and hyperlipidemia were more prevalent in the tamoxifen group (p=0.015 and p=0.020, respectively). After matching for age, type of surgery, and chronic disorder, all other variables were well balanced between the tamoxifen and control group, except for osteoporosis. A total of 95 patients on tamoxifen developed osteoporosis (95/1577, 6%), whereas 155 (155/1577, 9.8%) who were not receiving endocrine therapy developed osteoporosis (p<0.001). Osteoporosis remained significantly less prevalent in the tamoxifen group (**Table 1**).

**Table 1:** Comparison of clinical characteristics of patients with DCIS according to receipt of tamoxifen (osteoporosis diagnosis plus medication).

	Before matching			After matching		
	Patients not receiving tamoxifen, n=1,684 (%)	Patients receiving tamoxifen, n=2,970 (%)	*P*-value	Patients not receiving tamoxifen, n=1,577 (%)	Patients receiving tamoxifen, n=1,577 (%)	*P*-value
**Osteoporosis**			<0.001			<0.001
** No**	1519(90.20)	2779(93.57)		1422(90.17)	1482(93.98)	
** Yes**	165(9.80)	191(6.43)		155(9.83)	95(6.02)	
**Operation**			0.0006			0.9064
** Breast-conserving surgery**	1481(87.95)	2705(91.08)		1417(89.85)	1415(89.73)	
** Total mastectomy**	203(12.05)	265(8.92)		160(10.15)	162(10.27)	
**Diabetes**			0.015			0.7598
** No**	1523(90.44)	2617(88.11)		1427(90.49)	1432(90.81)	
** Yes**	161(9.56)	353(11.89)		150(9.51)	145(9.19)	
**Hypertension**			0.2933			0.4093
** No**	1352(80.29)	2346(78.99)		1276(80.91)	1294(82.05)	
** Yes**	332(19.71)	624(21.01)		301(19.09)	283(17.95)	
**Hyperlipidemia**			0.0203			0.8059
** No**	1258(74.70)	2125(71.55)		1181(74.89)	1175(74.51)	
** Yes**	426(25.30)	845(28.45)		396(25.11)	402(25.49)	
**COPD**			0.9101			0.4342
** No**	1644(97.62)	2901(97.68)		1544(97.91)	1550(98.29)	
** Yes**	40(2.38)	69(2.32)		33(2.09)	27(1.71)	
**CKD**			0.3338			>0.999
** No**	1667(98.99)	2948(99.26)		1570(99.56)	1570(99.56)	
** Yes**	17(1.01)	22(0.74)		7(0.44)	7(0.44)	
**LC**			0.1662			0.6872
** No**	1676(99.52)	2963(99.76)		1573(99.75)	1575(99.87)	
** Yes**	8(0.48)	7(0.24)		4(0.25)	2(0.13)	
**Heart failure**			0.2147			0.1956
** No**	1667(98.99)	2950(99.33)		1567(99.37)	1572(99.68)	
** Yes**	17(1.01)	20(0.67)		10(0.63)	5(0.32)	
**Age (year, mean ± SD)**	48.552 ± 10.414	49.215 ± 9.4	0.0308	48.984 ± 9.852	48.898 ± 9.713	0.8045

DCIS, ductal carcinoma in situ; COPD, chronic obstruction pulmonary disease; CKD, chronic kidney disease; LC, liver cirrhosis; SD, standard deviation.

### 3.3 Risk factors of osteoporosis

**Figure 2:**
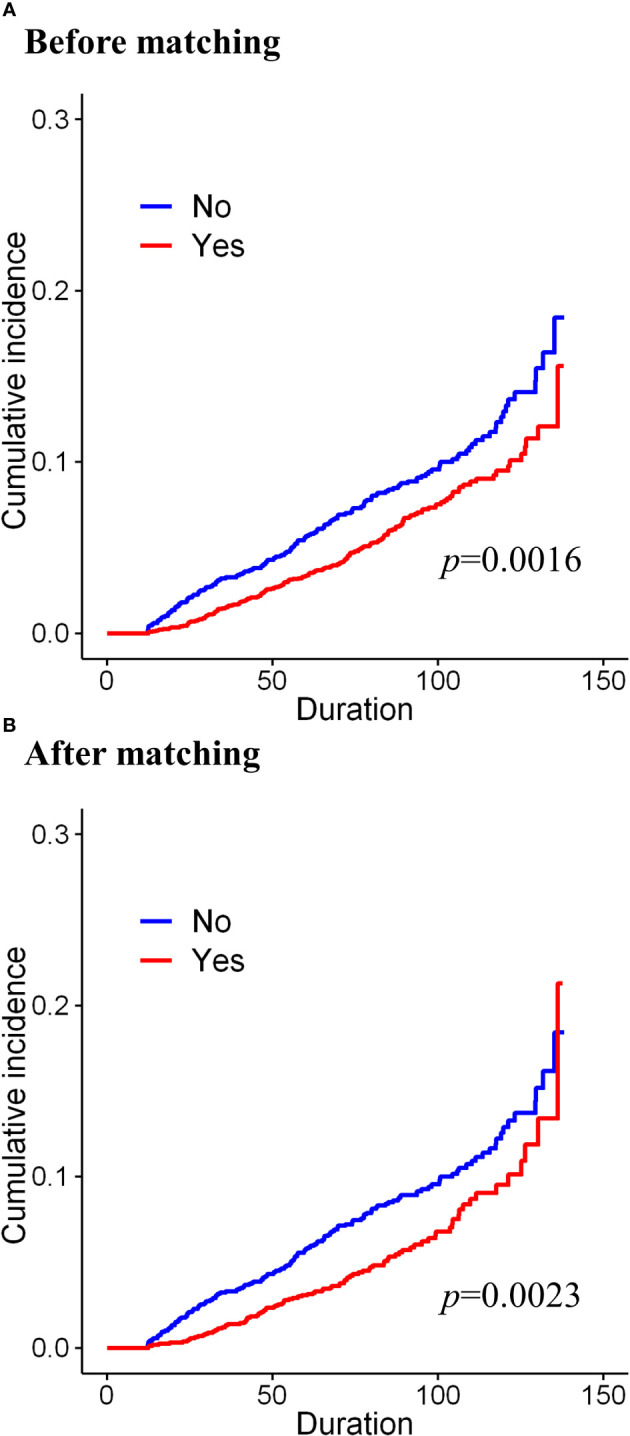
Cumulative incidence of osteoporosis according to the long-term use of tamoxifen **(A)** before matching and **(B)** after matching.

Before matching, during the median follow-up period of 87 months, the cumulative incidence of osteoporosis was significantly lower in the tamoxifen group (before matching, p=0.0016, **Figure 2A**). During the median follow-up of 88 months after matching, the cumulative incidence of osteoporosis was also significantly lower in the tamoxifen group than in the control group (after matching, p=0.0023, **Figure 2B**). Before matching, in the univariate Cox proportional hazard analysis, women who were on tamoxifen had a significantly lower risk of osteoporosis than tamoxifen users (**Table 2**; HR=0.715, 95% CI=0.580–0.881, p=0.0017). After adjusting for the type of surgery, age, and comorbidities, tamoxifen use was consistently associated with a lower risk of osteoporosis in the multivariable model (HR=0.690, 95% CI=0.559–0.851, p=0.0005). Along with tamoxifen use, age, hypertension, and hyperlipidemia were also associated with the development of osteoporosis from multivariable analysis (**Table 2**). In the univariable analysis, these factors remained significant in the matched cohort as well. Only hyperlipidemia and age were consistently significant from multivariate analysis (**Table 2**, HR=1.488, 95% CI=1.129–1.963, p=0.0049 and HR=1.086, 95% CI=1.072–1.101, p<0.001, respectively).

**Table 2:** Risk of developing osteoporosis from analyses using Cox proportional hazard models.

	Before matching				After matching			
	Univariate analysis		Multivariate analysis		Univariate analysis		Multivariate analysis	
	HR (95% CI)	*P* value	HR (95% CI)	*P*-value	HR (95% CI)	*P* value	HR (95% CI)	*P*-value
**Groups**		0.0017		0.0005		0.0025		0.0018
** Not receiving tamoxifen**	1		1		1		1	
** Receiving tamoxifen**	0.715(0.580-0.881)		0.690(0.559-0.851)		0.674(0.521-0.870)		0.664(0.513-0.858)	
**Operation**		0.5327		0.793		0.7594		0.8149
** BCS**	1		1		1		1	
** TM**	1.113(0.795-1.558)		0.956(0.680-1.342)		1.066(0.710-1.600)		0.952(0.633-1.433)	
**Diabetes**		0.0002		0.1228		0.0012		0.0803
** No**	1		1		1		1	
** Yes**	1.717(1.295-2.275)		0.789(0.584-1.066)		1.791(1.257-2.550)		0.715(0.491-1.041)	
**Hypertension**		<0.001		0.0225		<0.001		0.2075
** No**	1		1		1		1	
** Yes**	1.924(1.540-2.402)		0.735(0.564-0.958)		2.099(1.606-2.744)		0.817(0.596-1.119)	
**Hyperlipidemia**		<0.001		0.0003		<0.001		0.0049
** No**	1		1		1		1	
** Yes**	2.293(1.856-2.832)		1.531(1.215-1.928)		2.354(1.826-3.034)		1.488(1.129-1.963)	
**COPD**		0.0458		0.9809		0.8286		0.3796
** No**	1		1		1		1	
** Yes**	1.759(1.010-3.060)		0.993(0.571-1.728)		1.103(0.455-2.674)		0.682(0.291-1.601)	
**CKD**		0.6813		0.3313		0.9624		0.4596
** No**	1		1		1		1	
** Yes**	0.747(0.186-2.999)		0.539(0.155-1.874)		1.048(0.147-7.466)		0.543(0.108-2.739)	
**LC**		0.5963		0.4883		0.8593		0.9474
** No**	1		1		1		1	
** Yes**	0.472(0.029-7.592)		0.378(0.024-5.930)		1.286(0.080-20.763)		1.098(0.069-17.488)	
**Heart failure**		0.1916		0.8844		0.8814		0.2108
** No**	1		1		1		1	
** Yes**	1.801(0.745-4.356)		0.938(0.399-2.210)		0.861(0.121-6.138)		0.358(0.071-1.790)	
**Age**	1.078(1.068-1.088)	<0.001	1.083(1.071-1.095)	<0.001	1.079(1.068-1.091)	<0.001	1.086(1.072-1.101)	<0.001

HRs, hazard ratios; CIs, confidence intervals; BCS, breast conserving surgery; TM, total mastectomy; COPD, chronic obstruction pulmonary disease; CKD, chronic kidney disease; LC, liver cirrhosis.

### 3.4 Risk factors of osteoporosis by menopausal status

**Figure 3:**
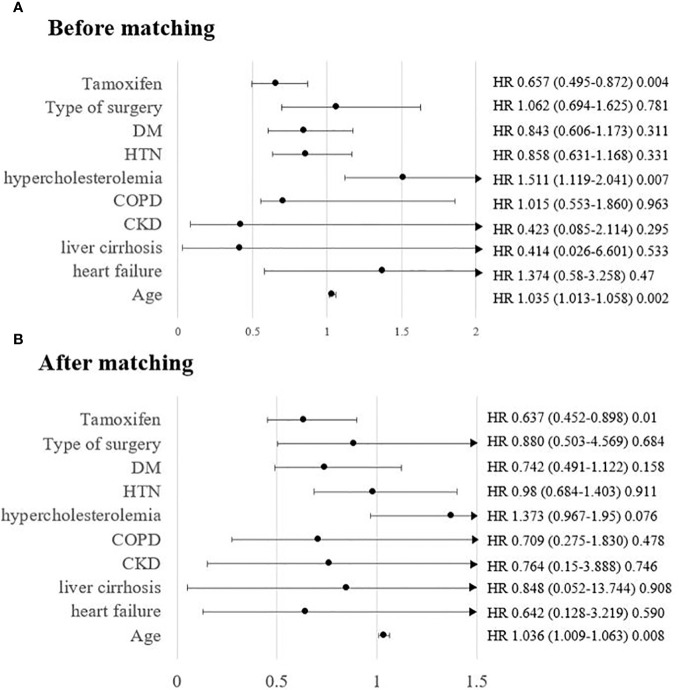
Risk of osteoporosis in patients taking tamoxifen in postmenopausal patients (age > 55 years) **(A)** before matching and **(B)** after matching.

We also carried out subgroup analysis by patient age. Since the HIRA registry does not provide information on the menopausal status, we subdivided the patients into three groups by age: <45, 45–55, and >55 years, representing the premenopausal, perimenopausal, and postmenopausal state, respectively. **Supplementary Tables 2-4** show the differences in variables between the groups according to tamoxifen use and age, before and after matching. There were few incidences of osteoporosis in the premenopausal group: 24 out of 1,553 patients and 11 out of 1,056 patients (**Supplementary Table 2**, 1.5% before matching and 1.0% after matching, respectively). The incidence of osteoporosis regardless of tamoxifen use increased gradually in the perimenopausal and postmenopausal groups from 6.8% and 7.6% (**Supplementary Table 3**, 135/1,978 before matching and 99/1,302 after matching, respectively) to 17.5% and 17.6% (**Supplementary Table 4**, 197/1,123 before matching and 140/796 after matching, respectively), respectively. Age-dependent subgroup analysis showed that the protective effect of tamoxifen was robust in the older age group (**Figure 3** and **Supplementary Table 5**, before matching and after matching, p=0.0009 and p=0.0015, respectively). The results of the univariate analysis showed that compared to other age groups, only the postmenopausal group showed a definite protective effect of tamoxifen (**Supplementary Figure 3**; before matching, HR=0.68, 95% CI=0.514–0.9, p=0.007, **Supplementary Figure 1**; after matching, HR=0.638, 95% CI=0.453–0.899, p=0.01). In the cohorts before and after matching, multivariable analysis revealed that tamoxifen was not associated with osteoporosis in premenopausal women (<45 years old) (**Supplementary Figure 4** [before matching, multivariable], HR=1.334, 95% CI=0.545–3.262, p=0.528, **Supplementary Figure 2** [after matching, multivariable], HR=0.705, 95% CI=0.199–2.494, p=0.587). In perimenopausal women, tamoxifen was significantly associated with a reduced risk of developing osteoporosis before matching (**Supplementary Figure 4**, HR=0.678, 95% CI=0.482–0.955, p=0.026). After adjusting for age and comorbidities, the protective effect persisted in perimenopausal women, but statistical significance was marginal (**Supplementary Figure 2**, HR=0.684, 95% CI=0.454–1.029, p=0.068). In postmenopausal women, the protective effect of tamoxifen was shown evidently both before and after matching. The multivariable analysis showed a consistent protective effect of tamoxifen before and after the matching (**Supplementary Figure 4**; before matching, HR=0.657, 95% CI=0.495–0.872, p=0.004; **Supplementary Figure 2**; after matching, HR=0.637, 95% CI=0.452–0.898, p=0.010, respectively). There was a 35% reduction in the risk of developing osteoporosis when the postmenopausal population was treated with tamoxifen. Age was the only significant factor in both the univariable and multivariable models.

## 4 Discussion

The findings from this nationwide cohort study of DCIS patients suggest that the use of tamoxifen is associated with a reduced risk of osteoporosis. The incidence of osteoporosis was similar between tamoxifen users and non-users younger than 45 years, presumably premenopausal women. However, in the older group of postmenopausal women, tamoxifen use seemed to have a beneficial effect as well as a low associated risk of osteoporosis.

Selective estrogen receptor modulators in breast cancer and DCIS patients, such as tamoxifen, act as partial ER agonists in the bone and are known for their protective effect (15–17). Previous studies have shown that postmenopausal breast cancer patients benefit from preserved bone density due to long-term tamoxifen use compared to their age-matched controls, even in the late menopause phase (15, 18).

Estradiol plays an important role in breast cancer development and progression. Premenopausal breast cancer survivors with hormone receptor-positive disease, accounting for approximately 70% of diagnosed breast tumors, suffer from adverse effects of long-term estradiol deprivation. Because endogenous estradiol is more potent than tamoxifen, tamoxifen acts as a partial antagonist in the bone, competing with estradiol for receptor binding, resulting in increased bone remodeling and bone loss (19). Approximately 35% of the overall population in our study belonged to the premenopausal group, and interestingly, our findings do not demonstrate an increased risk of osteoporosis in this group.

The adverse effects of tamoxifen on premenopausal women have been demonstrated in several studies. An NSABP P-1 study was conducted in high-risk women who had taken only tamoxifen (9). The difference from our study was that the endpoint was the incidence of fractures, while instances of subclinical osteoporosis not complicated with fracture were not considered. Furthermore, the patient characteristics were different from those of our sample, where the NSABP P-1 was a prevention trial that comprised healthy individuals who were at high risk of developing breast cancer. The difference in patient characteristics may explain the dissimilar results: tamoxifen reduced the risk of fractures in premenopausal patients in the NSABP P-1 trial, but fractures were not considered in the current study. Based on bone mineral density (BMD) measured by dual-energy X-ray absorptiometry (DEXA), tamoxifen led to 1.44% more annual bone loss than placebo. The difference between tamoxifen and placebo became prominent on sequential DEXA during the 3-year follow-up on both lumbar and hip BMD (20). Another case-control study of premenopausal women with early breast cancer treated with adjuvant chemotherapy showed similar results; at the 3-year follow-up, menstruating patients on tamoxifen showed increased bone loss (-4.6%) from the baseline BMD values (21). Nevertheless, these studies were based on small samples of premenopausal patients, and the patients had received other cancer treatments that could have influenced BMD.

A recent study in South Korea reported that the risk of osteoporosis in younger breast cancer patients with invasive breast cancer had not increased (22). In line with this, our results showed that there was no increased risk of osteoporosis in breast cancer survivors taking tamoxifen who were diagnosed in the premenopausal phase. Our sample consisted of only DCIS patients, all uniformly treated according to the national guidelines that assure 5-year treatment of tamoxifen, regardless of menopausal status in women with hormone receptor-positive *in situ* disease. Further, chemotherapy was spared as chemotherapy may have a direct and indirect effect on bone loss, mostly due to premature ovarian function failure (23–25). Lastly, we conducted propensity score matching using the type of surgery as a covariate, which is a surrogate for radiation therapy, to reduce the bias as much as possible.

Our study has several limitations due to the nature of the observational cohort. Health insurance claims data do not include specific data on lifestyle, anthropometric data, and socioeconomic status, all of which can influence the development of osteoporosis. Despite the fact that the bone mineral density was not known, patients taking medicine for osteoporosis can be interpreted as having a T score of ≤-2.5 because that is when the insurance covers the osteoporosis medication. Furthermore, we did not have the means to investigate adherence to tamoxifen. Detailed exposure assessment was limited and the median time between the duration of tamoxifen and development of osteoporosis could not be determined in this study. The current study examined the association between long-term tamoxifen use and osteoporosis, and other serious adverse events were not included. Of note, skeletal-related events represented by osteoporosis are the main reasons for reduced compliance; hence, clinicians can use the data from this study to promote compliance among their patients.

In conclusion, the long-term follow-up of a longitudinal, observational cohort of a homogeneously treated population demonstrated that tamoxifen had a protective effect on postmenopausal women, and the effect was equivocal in premenopausal women. These findings add to the evidence that can be used for addressing patients’ concerns about the risk of osteoporosis due to tamoxifen treatment.

## Data availability statement

All data generated or analyzed during this study are included in this research article and the **supplementary information files**. However, the original data is prohibited from being exported outside due to NHI policy. Requests to access the datasets should be directed to fayn@daum.net.

## Ethics statement

The studies involving humans were approved by Institutional Review Board of Seoul St. Mary’s Hospital (IRB number: KC22ZISI0340). All procedures performed in studies involving human participants were carried out in accordance with the ethical standards of the institutional and/or national research committee and with the 1964 Declaration of Helsinki and its later amendments or comparable ethical standards. The ethics committee/institutional review board waived the requirement of written informed consent for participation from the participants or the participants’ legal guardians/next of kin due to the retrospective study design.

## Author contributions

CY had full access to all of the data in the study and takes responsibility for the integrity of the data and the accuracy of the data analysis. Conceptualization, DK and CY; Data curation, HL and SJ; Funding acquisition, JO and CY; Investigation, DK, HL, SJ, and CY; Methodology, DK, OJ, HL, SJ, and CY; Resources, HL and SJ; Formal analysis, DK and CY; Supervision, WP; Writing-original draft, DK and CY. All authors contributed to the article and approved the submitted version.
