# Supplementary figures and images for: Association between tamoxifen and incidence of osteoporosis in Korean patients with ductal carcinoma in situ
Source: Front Oncol. 2024 Jan 8;13:1236188. doi: 10.3389/fonc.2023.1236188 (PMC10801186; doi:10.3389/fonc.2023.1236188)

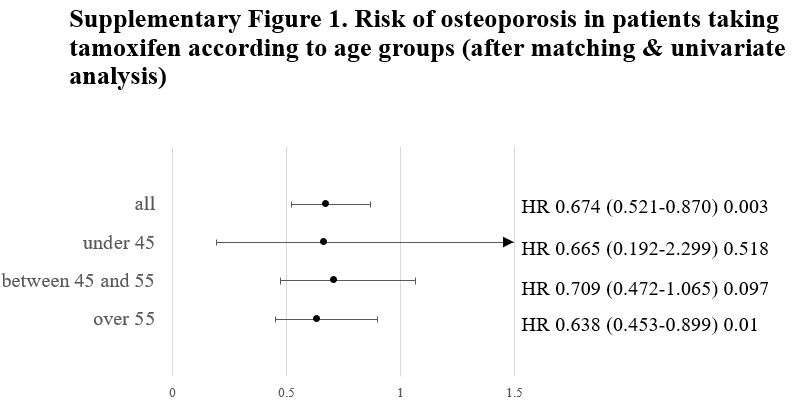

Supplement: Supplementary file 1 [file Image_1.tif]

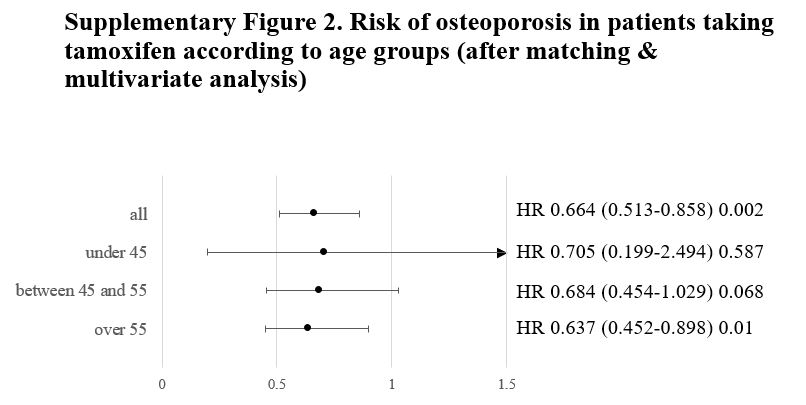

Supplement: Supplementary file 2 [file Image_2.tif]

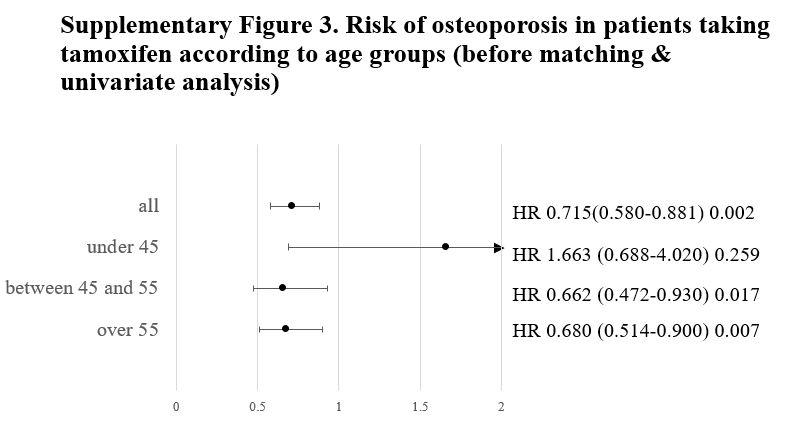

Supplement: Supplementary file 3 [file Image_3.tif]

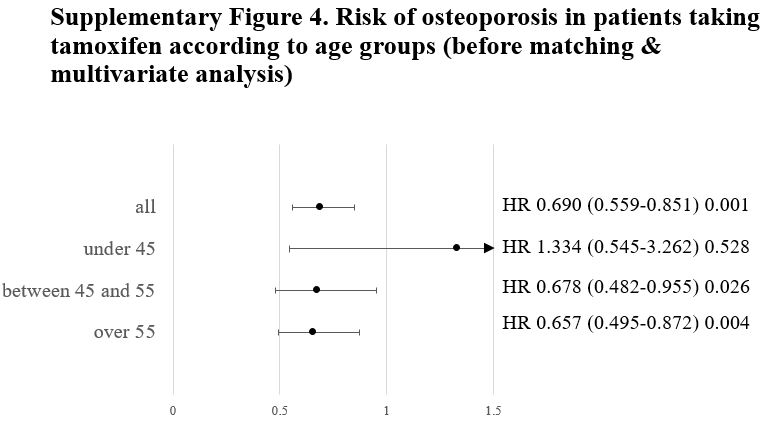

Supplement: Supplementary file 4 [file Image_4.tif]
