# Supplementary material for: Association between tamoxifen and incidence of osteoporosis in Korean patients with ductal carcinoma in situ
Source: Front Oncol. 2024 Jan 8;13:1236188. doi: 10.3389/fonc.2023.1236188 (PMC10801186; doi:10.3389/fonc.2023.1236188)
Supplement: Supplementary file 5 [file Table_1.docx]

**Supplementary Table 1. Prescription codes for medication**

| **Medication** | **Prescription code** |
| --- | --- |
| **Risedronate** | 442301ATB, 442302ATB , 442303ATB, 442330ATB, 500901ATB, 511200ATB |
| **Ibandronate** | 480301BIJ, 480302BIJ, 480303BIJ, 480304ATB, 480330BIJ |
| **Etidronate** | 147401ATB |
| **Pamidronate** | 207901ACS, 207902BIJ, 207930BIJ |
| **Alendronate** | 228301ATB, 228302ATB, 228303ALQ, 228303ATB, 228305ATB |
| **Zoledronic acid** | 629002BIJ, 420701BIJ, 420731BIJ, 420732BIJ, 420702BIJ, 420730BIJ |
| **Denosumab** | 629001BIJ |

**Supplementary Table 2. Comparison of clinical characteristics of patients with DCIS according to receipt of tamoxifen (premenopausal status, age younger than 45 years)**

|  | **Before matching** |  |  | **After matching** |  |  |
| --- | --- | --- | --- | --- | --- | --- |
|  | **Patients not receiving tamoxifen, n=594 (%)** | **Patients receiving tamoxifen, n=959 (%)** | ***P* value** | **Patients not receiving tamoxifen, n=528 (%)** | **Patients receiving tamoxifen, n=528 (%)** | ***P* value** |
| **Osteoporosis** |  |  | 0.356 |  |  | 0.363 |
| **No** | 587 (98.8) | 942 (98.2) |  | 521 (98.7) | 524 (99.2) |  |
| **Yes** | 7 (1.2) | 17 (1.8) |  | 7 (1.3) | 4 (0.8) |  |
| **Operation** |  |  | 0.288 |  |  | 0.908 |
| **Breast conserving surgery** | 543 (91.4) | 861 (89.8) |  | 487 (92.2) | 488 (92.4) |  |
| **Total mastectomy** | 51 (8.6) | 98 (10.2) |  | 41 (7.8) | 40 (7.6) |  |
| **Diabetes** |  |  | <0.001 |  |  | >0.999 |
| **No** | 589 (99.2) | 917 (95.6) |  | 523 (99) | 523 (99) |  |
| **Yes** | 5 (0.8) | 42 (4.4) |  | 5 (1) | 5 (1) |  |
| **Hypertension** |  |  | 0.342 |  |  | 0.498 |
| **No** | 571 (96.1) | 912 (95.1) |  | 508 (96.2) | 512 (97) |  |
| **Yes** | 23 (3.9) | 47 (4.9) |  | 20 (3.8) | 16 (3) |  |
| **Hyperlipidemia** |  |  | 0.12 |  |  | 0.499 |
| **No** | 530 (89.2) | 830 (86.5) |  | 471 (89.2) | 464 (87.9) |  |
| **Yes** | 64 (10.8) | 129 (13.5) |  | 57 (10.8) | 64 (12.1) |  |
| **COPD** |  |  | 0.775 |  |  | >0.999 |
| **No** | 589 (99.2) | 952 (99.3) |  | 526 (99.6) | 525 (99.4) |  |
| **Yes** | 5 (0.8) | 7 (0.7) |  | 2 (0.4) | 3 (0.6) |  |
| **CKD** |  |  | 0.334 |  |  | >0.999 |
| **No** | 592 (99.7) | 951 (99.1) |  | 528 (100) | 527 (99.8) |  |
| **Yes** | 2 (0.3) | 8 (0.9) |  | 0 (0) | 1 (0.2) |  |
| **LC** |  |  | >0.999 |  |  | >0.999 |
| **No** | 594 (100) | 958 (99.9) |  | 52800 | 528 (100) |  |
| **Yes** | 0 (0) | 1 (0.1) |  | 0 (0) | (0) |  |
| **Heart failure** |  |  | 0.159 |  |  | >0.999 |
| **No** | 591 (99.5) | 958 (99.9) |  | 527 (99.8) | 527 (99.8) |  |
| **Yes** | 3 (0.5) | 1 (0.1) |  | 1 (0.2) | 1 (0.2) |  |
| **Age (year, mean±SD)** | 37.95±5.23 | 39.73±4.35 | <0.001 | 38.8±4.68 | 38.76±4.71 | 0.906 |

COPD, chronic obstruction pulmonary disease; CKD, chronic kidney disease; LC, liver cirrhosis; SD, standard deviation

**Supplementary Table 3. Comparison of clinical characteristics of patients with DCIS according to receipt of tamoxifen (perimenopausal status, age between 45 and 55)**

|  | **Before matching** |  |  | **After matching** |  |  |
| --- | --- | --- | --- | --- | --- | --- |
|  | **Patients not receiving tamoxifen, n=660 (%)** | **Patients receiving tamoxifen, n=1,318 (%)** | ***P* value** | **Patients not receiving tamoxifen, n=651 (%)** | **Patients receiving tamoxifen, n=651 (%)** | ***P* value** |
| **Osteoporosis** |  |  | 0.001 |  |  | 0.016 |
| **No** | 598 (90.6) | 1245 (94.5) |  | 590 (90.6) | 613 (94.2) |  |
| **Yes** | 62 (9.4) | 73 (5.5) |  | 61 (9.4) | 38 (5.8) |  |
| **Operation** |  |  | 0.004 |  |  | 0.862 |
| **Breast conserving surgery** | 582 (88.2) | 1215 (92.2) |  | 577 (88.6) | 575 (88.3) |  |
| **Total mastectomy** | 78 (11.8) | 103 (7.8) |  | 74 (11.4) | 76 (11.7) |  |
| **Diabetes** |  |  | 0.26 |  |  | 0.655 |
| **No** | 614 (93) | 1207 (91.6) |  | 606 (93.1) | 610 (93.7) |  |
| **Yes** | 46 (7) | 111 (8.4) |  | 45 (6.9) | 41 (6.3) |  |
| **Hypertension** |  |  | 0.569 |  |  | 0.479 |
| **No** | 555 (84.1) | 1095 (83.1) |  | 552 (84.8) | 561 (86.2) |  |
| **Yes** | 105 (15.9) | 223 (16.9) |  | 99 (15.2) | 90 (13.8) |  |
| **Hyperlipidemia** |  |  | 0.242 |  |  | 0.636 |
| **No** | 511 (77.4) | 989 (75) |  | 508 (78) | 515 (79.1) |  |
| **Yes** | 149 (22.6) | 329 (25) |  | 143 (22) | 136 (20.9) |  |
| **COPD** |  |  | 0.725 |  |  | 0.39 |
| **No** | 647 (98) | 1295 (98.3) |  | 638 (98) | 642 (98.6) |  |
| **Yes** | 13 (2) | 23 (1.7) |  | 13 (2) | 9 (1.4) |  |
| **CKD** |  |  | 0.119 |  |  | >0.999 |
| **No** | 653 (98.9) | 1313 (99.6) |  | 650 (99.9) | 649 (99.7) |  |
| **Yes** | 7 (01.1 | 5 (0.4) |  | 1 (0.1) | 2 (0.3) |  |
| **LC** |  |  | 0.231 |  |  | 0.499 |
| **No** | 656 (99.4) | 1315 (99.8) |  | 649 (99.7) | 651 (100) |  |
| **Yes** | 4 (0.6) | 3 (0.2) |  | 2 (0.3) | (0) |  |
| **Heart failure** |  |  | >0.999 |  |  | 0.124 |
| **No** | 656 (99.4) | 1311 (99.5) |  | 647 (99.4) | 651 (100) |  |
| **Yes** | 4 (0.6) | 7 (0.5) |  | 4 (0.6) | 0 (0) |  |
| **Age (year, mean±SD)** | 49.39±2.775 | 49.11±2.699 | 0.03 | 49.36±2.752 | 49.33±2.732 | 0.725 |

COPD, chronic obstruction pulmonary disease; CKD, chronic kidney disease; LC, liver cirrhosis; SD, standard deviation

**Supplementary Table 4. Comparison of clinical characteristics of patients with DCIS according to receipt of tamoxifen (postmenopausal status, age greater than 55)**

|  | **Before matching** |  |  | **After matching** |  |  |
| --- | --- | --- | --- | --- | --- | --- |
|  | **Patients not receiving tamoxifen, n=430 (%)** | **Patients receiving tamoxifen, n=693 (%)** | ***P* value** | **Patients not receiving tamoxifen, n=398 (%)** | **Patients receiving tamoxifen, n=398 (%)** | ***P* value** |
| **Osteoporosis** |  |  | 0.001 |  |  | 0.002 |
| **No** | 334 (77.7) | 592 (85.4) |  | 311 (78.1) | 345 (86.7) |  |
| **Yes** | 96 (22.3) | 101 (14.6) |  | 87 (21.9) | 53 (13.3) |  |
| **Operation** |  |  | <0.001 |  |  | 0.911 |
| **Breast conserving surgery** | 352 (82.8) | 629 (90.8) |  | 353 (88.7) | 352 (88.4) |  |
| **Total mastectomy** | 74 (17.2) | 64 (9.2) |  | 45 (11.3) | 46 (11.6) |  |
| **Diabetes** |  |  | 0.232 |  |  | 0.935 |
| **No** | 320 (74.4) | 493 (71.1) |  | 298 (74.9) | 299 (75.1) |  |
| **Yes** | 110 (25.6) | 200 (28.9) |  | 100 (25.1) | 99 (24.9) |  |
| **Hypertension** |  |  | 0.236 |  |  | 0.721 |
| **No** | 226 (52.6) | 339 (48.9) |  | 216 (54.3) | 221 (55.5) |  |
| **Yes** | 204 (47.4) | 354 (51.1) |  | 182 (45.7) | 177 (44.5) |  |
| **Hyperlipidemia** |  |  | 0.039 |  |  | 0.671 |
| **No** | 217 (50.5) | 306 (44.2) |  | 202 (50.8) | 196 (49.2) |  |
| **Yes** | 213 (49.5) | 387 (55.8) |  | 196 (49.2) | 202 (50.8) |  |
| **COPD** |  |  | 0.713 |  |  | 0.594 |
| **No** | 408 (94.9) | 654 (94.4) |  | 380 (95.5) | 383 (96.2) |  |
| **Yes** | 22 (5.1) | 39 (5.6) |  | 18 (4.5) | 15 (3.8) |  |
| **CKD** |  |  | 0.454 |  |  | 0.525 |
| **No** | 422 (98.1) | 684 (98.7) |  | 392 (98.5) | 394 (99) |  |
| **Yes** | 8 (1.9) | 9 (1.3) |  | 6 (1.5) | 4 (1) |  |
| **LC** |  |  | 0.438 |  |  | >0.999 |
| **No** | 426 (99.1) | 690 (99.6) |  | 396 (99.5) | 396 (99.5) |  |
| **Yes** | 4 (0.9) | 3 (0.4) |  | 2 (0.5) | 2 (0.5) |  |
| **Heart failure** |  |  | 0.485 |  |  | >0.999 |
| **No** | 420 (97.7) | 681 (98.3) |  | 393 (98.7) | 394 (99) |  |
| **Yes** | 10 (2.3) | 12 (1.7) |  | 5 (1.3) | 4 (1) |  |
| **Age (year, mean±SD)** | 61.91±6.36 | 62.55±6.02 | 0.09 | 61.84±6.31 | 61.63±5.78 | 0.627 |

COPD, chronic obstruction pulmonary disease; CKD, chronic kidney disease; LC, liver cirrhosis; SD, standard deviation

**Supplementary Table 5. Risk of developing osteoporosis from analyses using Cox proportional hazard models (age greater than 55)**

|  | **Before matching** | | | | **After matching** | | | |
| --- | --- | --- | --- | --- | --- | --- | --- | --- |
|  | **Univariate analysis** | | **Multivariate analysis** | | **Univariate analysis** | | **Multivariate analysis** | |
|  | **HR (95% CI)** | ***P* value** | **HR (95% CI)** | ***P* value** | **HR (95% CI)** | ***P* value** | **HR (95% CI)** | ***P* value** |
| **Groups** |  | 0.007 |  | 0.004 |  | 0.01 |  | 0.01 |
| **Receiving tamoxifen** | 1 |  | 1 |  | 1 |  | 1 |  |
| **Not receiving tamoxifen** | 0.680 (0.514-0.9) |  | 0.657 (0.495-0.872) |  | 0.638 (0.453-0.899) |  | 0.637 (0.452-0.898) |  |
| **Operation** |  | 0.609 |  | 0.781 |  | 0.505 |  | 0.684 |
| **BCS** | 1 |  | 1 |  | 1 |  | 1 |  |
| **TM** | 1.116 (0.733-1.698) |  | 1.062 (0.694-1.625) |  | 0.824 (0.465-1.458) |  | 0.888 (0.503-1.569) |  |
| **Diabetes** |  | 0.67 |  | 0.311 |  | 0.425 |  | 0.158 |
| **No** | 1 |  | 1 |  | 1 |  | 1 |  |
| **Yes** | 0.933 (0.678-1.283) |  | 0.843 (0.606-1.173) |  | 0.850 (0.570-1.267) |  | 0.742 (0.491-1.122) |  |
| **Hypertension** |  | 0.810 |  | 0.331 |  | 0.601 |  | 0.91 |
| **No** | 1 |  | 1 |  | 1 |  | 1 |  |
| **Yes** | 1.035 (0.783-1.368) |  | 0.858 (0.631-1.168) |  | 1.093 (0.784-1.523) |  | 0.98 (0.684-1.403) |  |
| **Hyperlipidemia** |  | 0.019 |  | 0.007 |  | 0.089 |  | 0.076 |
| **No** | 1 |  | 1 |  | 1 |  | 1 |  |
| **Yes** | 1.404 (1.056-1.867) |  | 1.511 (1.119-2.041) |  | 1.336 (0.957-1.867) |  | 1.373 (0.967-1.950) |  |
| **COPD** |  | 0.800 |  | 0.963 |  | 0.437 |  | 0.476 |
| **No** | 1 |  | 1 |  | 1 |  | 1 |  |
| **Yes** | 1.082 (0.589-1.989) |  | 1.015 (0.553-1.860) |  | 0.674 (0.249-1.823) |  | 0.709 (0.275-1.830) |  |
| **CKD** |  | 0.309 |  | 0.295 |  | 0.677 |  | 0.746 |
| **No** | 1 |  | 1 |  | 1 |  | 1 |  |
| **Yes** | 0.361 (0.051-2.573) |  | 0.423 (0.085-2.114) |  | 0.658 (0.092-4.697) |  | 0.764 (0.150-3.888) |  |
| **LC** |  | 0.309 |  | 0.295 |  | 0.677 |  | 0.746 |
| **No** | 1 |  | 1 |  | 1 |  | 1 |  |
| **Yes** | 0.429 (0.027-6.938) |  | 0.414 (0.026-6.601) |  | 0.808 (0.050-13.151) |  | 0.848 (0.052-13.744) |  |
| **Heart failure** |  | 0.512 |  | 0.47 |  | 0.571 |  | 0.59 |
| **No** | 1 |  | 1 |  | 1 |  | 1 |  |
| **Yes** | 1.346 (0.554-3.269) |  | 13741 (0.580-3.258) |  | 0.566 (0.079-4.049) |  | 0.642 (0.128-3.219) |  |
| **Age** | 1.031 (1.010-1.052) | 0.003 | 1.035 (1.013-1.058) | 0.002 | 1.03 (1.005-1.055) | 0.017 | 1.036 (1.009-1.063) | 0.008 |

HRs, hazard ratios; CIs, confidence intervals; BCS, breast conserving surgery; TM, total mastectomy; COPD, chronic obstruction pulmonary disease; CKD, chronic kidney disease; LC, liver cirrhosis
